# Correlative iPALM and SEM resolves virus cavity and Gag lattice defects in HIV virions

**DOI:** 10.1007/s00249-018-1324-0

**Published:** 2018-07-24

**Authors:** Meike Pedersen, Shirin Jamali, Ipsita Saha, Rainer Daum, Mourad Bendjennat, Saveez Saffarian

**Affiliations:** 1 FEI Deutschland GmbH, Frankfurt, Germany; 2 Dept. of Physics and Astronomy, University of Utah, Salt Lake City, USA; 3 Center for Cell and Genome Science, University of Utah, Salt Lake City, USA; 4 Dept. of Biology, University of Utah, Salt Lake City, USA

**Keywords:** iPALM, SEM, HIV, Gag, CLEM

## Abstract

Interferometric Photo-Activation-Localization-Microscopy (iPALM) localizes single fluorescent molecules with 20 nm lateral and 10 nm axial resolution. We present a method utilizing glass coverslip lithography for correlative imaging between iPALM and scanning electron microscopy (SEM). Using iPALM on HIV Gag-Dendra virus-like particles (VLPs) we localized the position of HIV Gag proteins. Based on these localizations we reconstructed the central cavity of the VLPs along with imperfections within the HIV Gag lattice. The SEM images and iPALM images overlap and show imaging from single VLPs immobilized on glass coverslips. The localization of many HIV proteins including accessory proteins and Gag-Pol remains unknown, we discuss how the specificity of iPALM coupled with SEM has the potential for resolving more of HIV proteins.

**Electronic supplementary material:**

The online version of this article (10.1007/s00249-018-1324-0) contains supplementary material, which is available to authorized users.

## Introduction

Resolution of fluorescence microscopy is limited by diffraction; however, the advantage of fluorescence is that fluorescent tags are very specific. The position of a single fluorescent molecule can be determined based on the center of its diffraction limited image with nanometer precision (Thompson et al. 2002). It is therefore possible to reconstruct an image by activating the molecules one at a time and obtaining their position with nanometer precision. This principle was explored in photoactivatable localization microscopy [PALM, (Kaksonen and Drubin 2006)] fluorescence photoactivatable localization microscopy [fPALM, (Hess et al. 2006)] and stochastic optical reconstruction microscopy [STORM, (Rust et al. 2006)] to achieve in plane resolutions of 20 nm. The axial resolution of these techniques can be extended either through introduction of astigmatism associated with the out of plane images (Huang et al. 2008) or using Biplane imaging (Juette et al. 2008) both of which report an axial resolution of 50 nm and in focus resolution of 20 nm.

iPALM (Shtengel et al. 2009) collects the full wavefront of each photon emitted from the sample using two high NA objective. The two wave fronts collected from the top and bottom of the sample are passed through a three-phase beam splitter which introduces three 120° phase shifts resulting in three separate images with different interference between the two wave fronts. The three images are phase-shifted so that a *Z* distance of 80 nm results in full disappearance of signal from one image and its maximum appearance on the second image creating a sensitivity to distances as small as 8 nm along the axial direction depending on the number of photons collected from the sample. For typical fluorescence proteins a resolution below 20 nm in plane and 10 nm axial has been reported (Shtengel et al. 2009).

The main limitation of single molecule localization based high resolution microscopy techniques including iPALM, however, is the limited amount of information which can be obtained from the sample based on positions of only one kind of protein. Therefore, it is critical to visualize the rest of the sample with a different mode of imaging as shown previously for AFM (Hodges et al. 2013; Hodges and Saffarian 2014) and electron microscopy (Kopek et al. 2017).

In particular iPALM has the resolution to visualize the position of proteins within the capsid of purified virions. These virions are easily immobilized on glass surfaces and these surfaces can be efficiently imaged through deposition of a thin layer of metal using scanning electron microscopy. Here we have designed a method for correlative light and electron microscopy in which immobilized virions can be first visualized using iPALM and then coated with thin layers of metal and visualized in SEM. Specific lithography patterns on the glass allow registration of same areas in between SEM and iPALM, while 20 nm Gold nanoparticles embedded in the sample serve as guide posts which allow aligning the SEM and iPALM images.

HIV Gag alone is sufficient to create fully formed vesicles that bud into the extracellular space as virus like particles (VLPs) (similar diameter to full length HIV ~ 120 nm in diameter) (Gheysen et al. 1989). The Gag proteins incorporated within the VLP form a hexagonal lattice along the inside leaflet of the virus-like particle with defects to accommodate the virion’s curvature (Carlson et al. 2008). Approximately 2000 copies of Gag are present in purified wild-type HIV virions. The defects within the lattice of HIV Gag on the inside of HIV virions have been so far visualized using cryo-EM tomography (Briggs et al. 2004, 2009; Carlson et al. 2008, 2010); however, no optical measurements have been done to resolve these lattice arrangements using optical high resolution microscopy. Here we report 3D measurements of Gag-Dendra proteins within purified single virus-like particles which show both presence of the viral cavity as well as defects within the observed HIV Gag cavity.

## Materials and methods

### Lithography of glass coverslips

Optical lithography mask was prepared using Heidelberg MicroPG 101 Pattern Generator. The mask was developed with AZ developer 1:1 (made by AZ Electronic Materials USA Corp. 70 Meister Ave., Somerville, NJ 08876) for 45 s and the mask cleaning took place using a spin rinse dryer (SRD). The mask design pattern and sizes were verified by optical microscopy. The mask was placed in a chromium etch 1020AC (made by Transese Company Inc., 10 Electronic Avenue, Danvers, MA 01923) for 2.5 min, cleaned in DI water for 2 min, and then cleaned in an SRD.

### Cleaning coverslip glasses was done in two different ways

The first approach was using acetone/isopropanol (IPA) sonication for 5 min each and drying with N_2_. The second one was dipping the glass in BOE (buffered oxide etch) for few seconds followed by DI water rinse and dry with N_2_. The final result of fabrication with the first cleaning method was more successful as it produced better contrast between the pattern and the glass while imaging.

After cleaning steps the glasses were heated on hot plate at 120 °C for 10 min and then they cool down to room temperature after few minutes.

### Photolithography

Coverslips were spin-coated with hexamethyldisilazane (HMDS)/xylene (20:80) at 3000 rpm for 60 s to improve adhesion of photoresist to surface. After this step positive photoresist was spin coat S1813 (made by Shipley Company, 455 Forest St., Marlborough, Massachusetts 01752) at 3000 rpm for 60 s followed by soft bake using a hot plate at 110 °C for 1 min. The coverslip alignment with lithography mask was done using a Suss MA1006 aligner in hard contact mode. The coverslips were then exposed to UV light for 10 s and dipped into AZ developer 1:1 (made by AZ Electronic Materials USA Corp. 70 Meister Ave., Somerville, NJ 08876) for 45 s. Coverslips were then rinsed in DI water and dried with N_2_ and post baked using a hot plate at 110 °C for 2 min.

### Nano pattern was obtained by wet etching process

A BOE (buffered oxide etch or buffered HF) was used to etch the pattern for 3 min. This step was followed by thorough rinse with DI water. The etch quality was checked using an optical microscope and a profilometer to measure etch depth of 250 nm. Finally, the photoresist was removed by acetone and isopropanol (IPA) sonication for few minutes.

### HIV Gag VLP purification and deposition on glass

Humanized Gag was produced as previously described (Bendjennat and Saffarian 2016; Kofman et al. 2003) Dendra2 was fused in the Gag ORF after Gag-p6 as previously described (Ku et al. 2013). 293T cells were grown in complete DMEM medium under standard conditions. Gag-Dendra2 plasmids were transfected into 293T cells using standard CaPO_4_ precipitation technique. Both cells and media were collected for analysis. VLPs were pelleted from cell supernatants by centrifugation for 2 h through 10% (w/v) sucrose cushion at 15,000×*g*. Final VLP samples were re-suspended in PBS.

For sample preparation the lithography coverslips were sonicated for 30 min in ethanol followed by mQ water, followed by 1 M NaOh and again mQ water. Coverslips were dried under a N_2_ stream. 20 µl of VLP suspension in PBS was sandwiched between two coverslips and incubated for 10 min. Coverslips were then separated and rinsed with 10 ml of PBS at which point they were sandwiched with a clean coverslip on top and moved to iPALM.

### iPALM measurements

iPALM data were collected on a prototype setup (Thermo Fisher Scientific) using two Nikon 60× TIRF objectives, NA 1.46 and a custom three-way beam splitter as previously described (Shtengel et al. 2009). Fluorophores were excited using a LightHub Laser Line Combiner (405, 488, 562 and 638 nm) (Omicron) coupled to a Polytrope/Yanus TIRF excitation system (Thermo Fisher Scientific) with a single-mode fiber. Fluorescence was detected on three Hamamatsu Orca Flash 4 sCMOS cameras.

Raw data acquisition of iPALM consisted of two steps: Calibration of the setup using permanent fluorescent fiducials (20 nm Gold nanoparticles), and the experimental run detecting fluorescent events for 3D localization. During calibration the photons from the permanent fluorescing Gold nanoparticles were collected from the top and bottom objectives. The calibration procedure contained a well defined stepwise axial movement of the sample in between the two objectives (*z* stack at typically 8 nm steps with 101 planes). At each of the planes the fluorescence of the fiducial passes through a three-phase beam splitter. Monitoring the intensity change of these fiducials during the *z* stack reveals three separate interferograms with 120° phase shift on the three cameras. The raw data of this calibration were used to generate a look-up table for data post processing of the blinking events of the fluorescent proteins of the sample during an iPALM experiment.

During iPALM image acquisition the fluorescence intensity of blinking proteins was collected from 10,000 to 100,000 image sets. An image set contained three camera images. Due to the self-interference of photons the three-phase beam splitter converts the phase information into an intensity interference pattern registered on the three cameras. The relative intensities of the three images captured simultaneously on the three cameras contain the 3D information of every event. The signal to noise of the calibration interference patterns and single events of the proteins allow localization of their position with an axial resolution of typically *σ*_*z*_ < 10 nm. The localization precision dependents on the fluorescence emission wavelength of the fluorophore, and the number of emitted photons per fluorophore (Shtengel et al. 2009).

### Sample preparation for SEM

After completion of the iPALM experiments, the sandwiched coverslips were separated using a razor blade. The bottom coverslip which contained the lithographic patterns was then washed using 30 ml of mQ water and dried by side contact with filter paper. The dried coverslip was then placed in the chamber of an Emitech Sputtering machine and pumped until low vacuum conditions were achieved. 60 s of Gold Cadmium deposition was conducted with a calibrated deposition of 15 nm. After sputtering, the sample was removed from the vacuum chamber and stored in a closed box to protect from dust accumulation.

### SEM and correlation with iPALM

SEM imaging was performed on FEI Helios 650. DIC images were loaded in Maps for correlative microscopy (Thermo Scientific) and used to identify regions of interest on a Helios SEM (Thermo Scientific). For fine alignment between reconstructed iPALM images and SEM images Gold fiducials were used as markers in Maps. The final SEM image was reconstructed by creating a high resolution carpet image from six independent images with 10% overlapping scans as shown in Fig. 2. Each scan was performed using voltage of 2 keV, TLD detector and 2000× magnification resulting in a pixel size of 4 nm.

### iPALM data analysis and SEM

iPALM data were rendered in 3D using PeakSelector (Gleb Shtengel and Harald Hess, Howard Hughes Medical Institute) as described in (Shtengel et al. 2009). Briefly interference patterns were calibrated using *Z*-stacks recorded from 20 nm Gold nanoparticles (Nanopartz) located in the observed region. Gold nanoparticles were also used to correct for drift in *X*, *Y* and *Z*.

## Results

### Design of the workflow for SEM-iPALM correlative light and electron microscopy (CLEM)

**Fig. 1:**
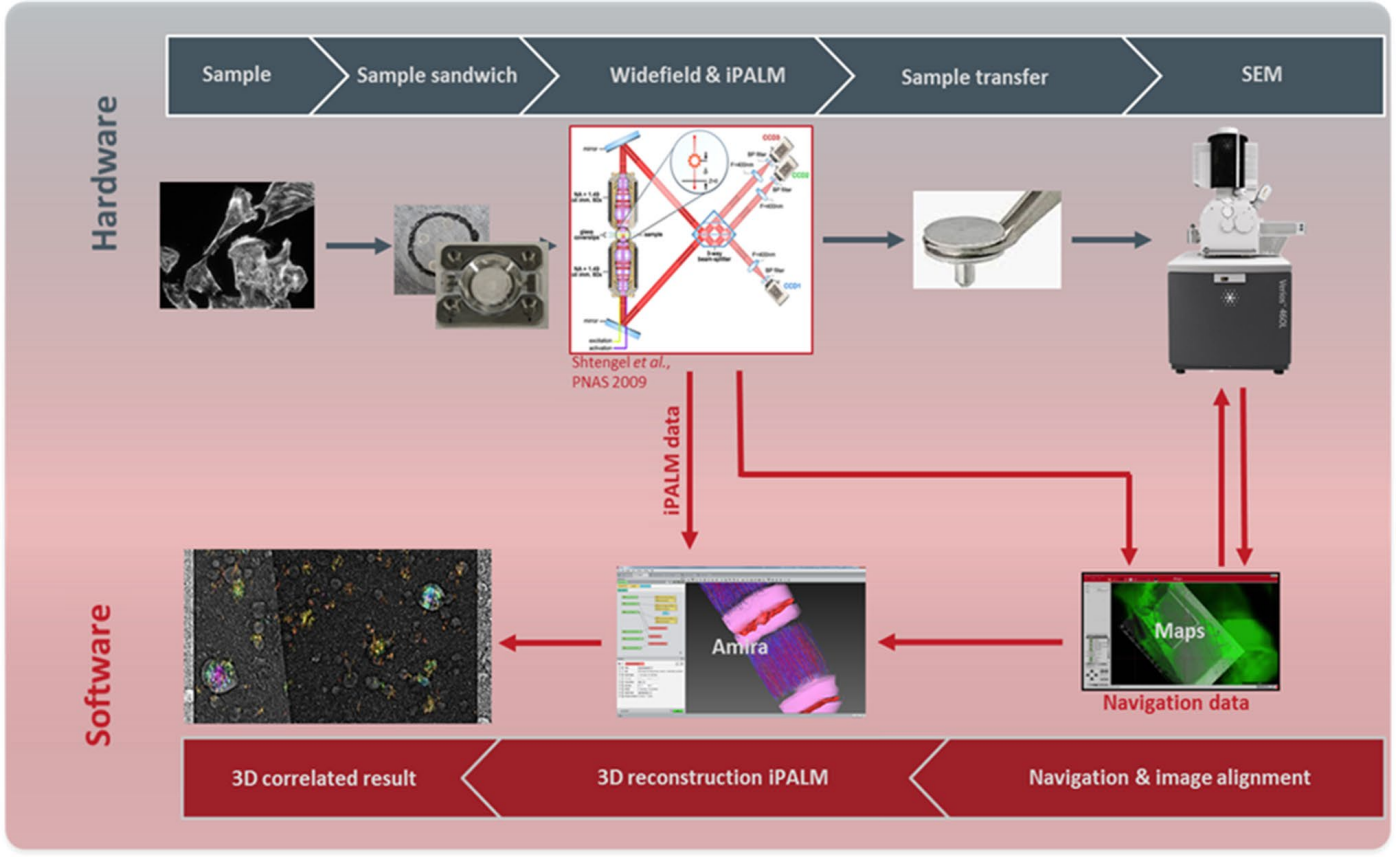
Workflow of iPALM-SEM CLEM microscopy. The sample is imaged by sandwiching between two glass coverslips using iPALM microscopy and DIC imaging. Glass coverslips are then separated and coated with 15 nm of Gold Cadmium before imaging by SEM. MAPS software is then used to alight SEM images with DIC images acquired during iPALM. Final alignment of SEM and iPALM is achieved through overlapping 20 nm Gold nanoparticles clearly visible in both iPALM and SEM

iPALM works best when the two high NA objectives are sandwiching the sample between two coverslips. The coverslips therefore provide a natural registration for correlating iPALM and electron microcopy. It is convenient to image the coverslips with the bound samples using SEM after the iPALM imaging on the sample are complete. As shown in Fig. 1, we have constructed a workflow to achieve this objective during CLEM experiments. The coverslips used during the experiments have lithography patterns as shown in Figure S1 (see “Materials and methods” section). These lithography patterns are visible by DIC imaging. iPALM imaging is computationally heavy and therefore in our design was limited to an area of 30 µm × 30 µm on the coverslips. During the experiments, 20 nm Gold nanoparticles were mixed with HIV Gag-Dendra VLPs before deposition on coverslips. A suitable area of the sample was found which was near a distinct lithography mark on the coverslip and contained more than three Gold nanoparticles within the imaging area of the iPALM. As shown in Fig. 1, a DIC image of the general area of the iPALM was collected before iPALM data acquisition. After iPALM imaging, the coverslips were separated from each other and a 15 nm layer of Gold Cadmium was sputtered on the sample before taking the sample for SEM. FEI MAPS software was used to find the iPALM area for further SEM imaging using the previously collected DIC image as a guide. Once the general area of the iPALM imaging on the glass coverslip was identified with SEM, a detailed scan of 4 nm/pixel was commenced which covered the full area of the iPALM imaging. The iPALM and SEM images were then correlated as detailed below.

### Correlating the general area of imaging between SEM and iPALM

**Fig. 2:**
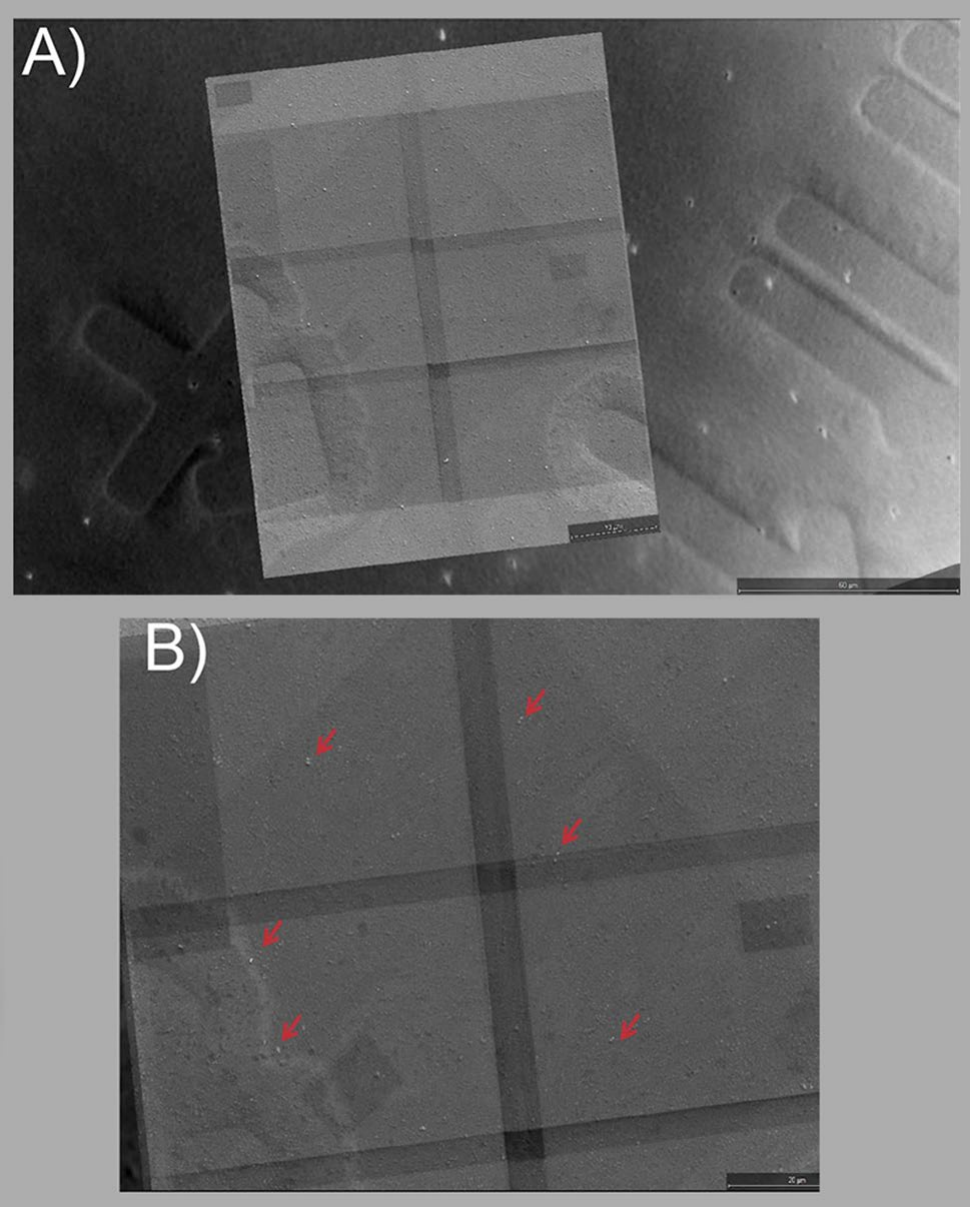
Initial alignment of SEM and iPALM microscopy based on DIC imaging. **a** Glass coverslips with lithographic markers etched on the surface of the glass. These Gold nanoparticles allow easy identification of an area of interest in SEM as well as DIC and iPALM microscopy. After deposition of sample DIC microscopy is used to identify a suitable region of interest for iPALM which will cover an area of 30 × 30 µm. The selected iPALM ROI should satisfy two criteria of having as many > 3 Gold nanoparticles as well as being at a convenient location to the nano-lithographic surface features of the glass coverslips. Once such an area is found, the iPALM data are collected and DIC images of the ROI are saved for CLEM. **b** A high resolution (4 nm/pixel) SEM scan is performed which encompasses the ROI previously measured in iPALM. Gold nanoparticles are identified in the ROI based on their high contrast in SEM. Scale bar represents 10 µm

The glass coverslip used during iPALM imaging has a 2-inch diameter. It is practically impossible to find the exact area of iPALM imaging (30 µm × 30 µm) in SEM without relying on specific markers etched on the coverslip surface. As shown in Fig. 2, the DIC images of the general iPALM area need to contain enough details of etched markers to allow identifying the same area in SEM. While 20 nm Gold nanoparticles are below diffraction limit, they are still visible in DIC imaging and therefore the random position of these particles along with the surface marks are used to locate the general iPALM area during SEM imaging. Once this area is identified, a 4 nm/pixel detailed scan is performed by SEM which covers the full 30 µm × 30 µm area of iPALM imaging. As shown in Fig. 2b, the Gold nanoparticles are clearly visible in both SEM and DIC imaging as shown by red arrows.

### Triangulating the sample using Gold nanoparticles visualized in iPALM and SEM

**Fig. 3:**
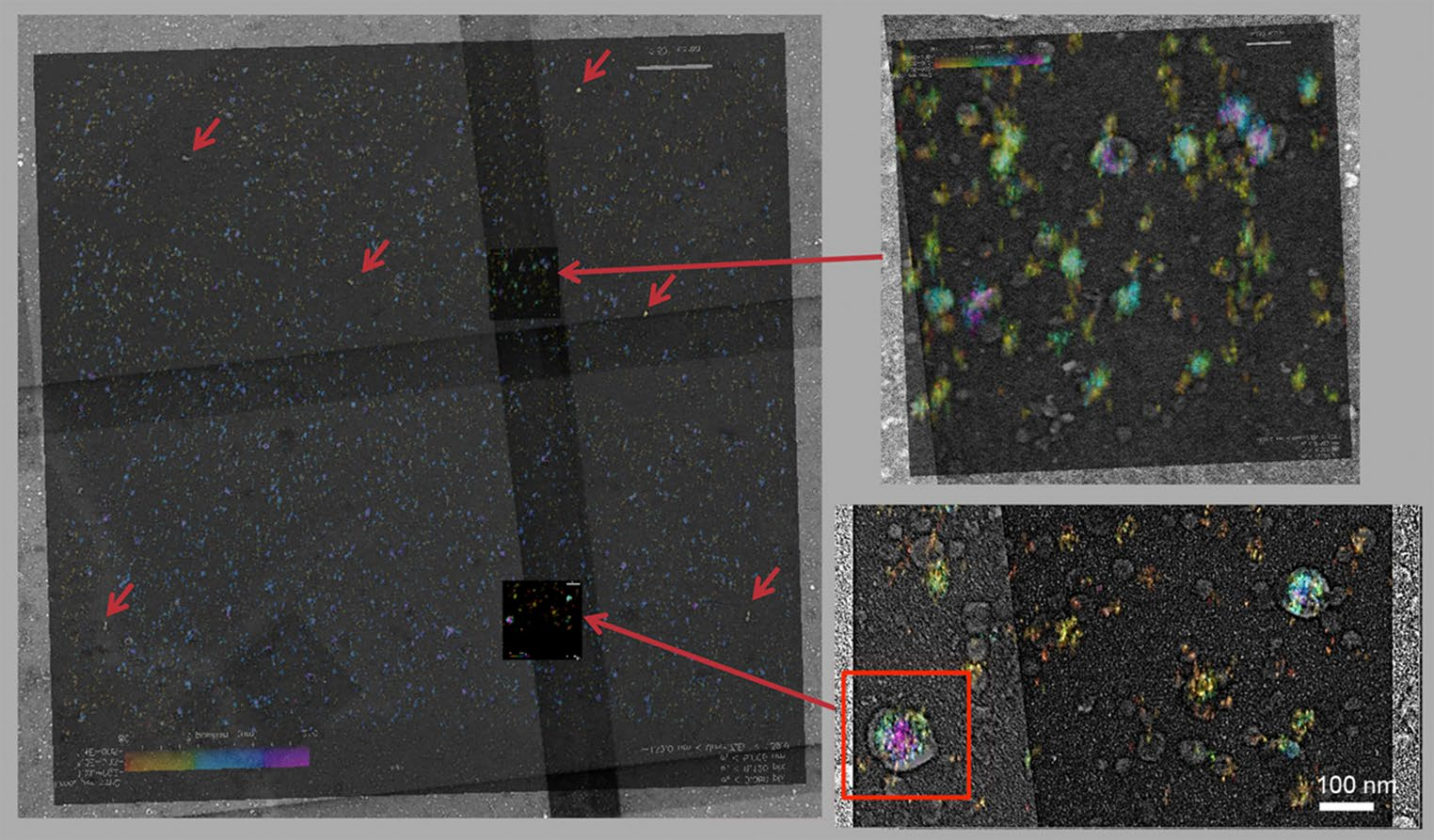
Large scale alignment of iPALM and SEM imaging using Gold nanoparticles. Superposition of iPALM and SEM based on identified Gold nanoparticles. An HIV VLP is identified shown by the red box with superimposed SEM and iPALM data. There are clear areas around each Gold nanoparticle where no iPALM localization is found due to the overwhelming signal from the Gold nanoparticles. **b**, **c** Zoom-in views of the overlap between SEM and iPALM. As shown in (**b**) not all iPALM localizations are correlating with observed VLPs in SEM. The iPALM localizations devoid of VLPs represent presence of residual Gag-Dendra molecules immobilized on glass along with VLPs. **c** An overlap between SEM image of a VLP and its iPALM localization

iPALM data were acquired for 10,000 frames in which Dendra molecules were localized in each frame and their 3D position recorded. Activated Dendra molecules photobleach within a few frames and therefore their localization is limited to the few frames in which the Dendra signal appears. The Gold nanoparticles, however, fluoresce with similar intensity in all acquired frames. Therefore, as shown in Fig. 3, the Gold nanoparticles in iPALM are clearly identified as shown with red arrows. Please note the hollow area around each Gold nanoparticle in the iPALM image, as any molecule too close to the Gold nanoparticles would not be localized due to overlap with the point spread function of the Gold nanoparticles. Gold nanoparticles also exhibited a large contrast in the SEM imaging due to conductance of Gold.

SEM images and iPALM images are overlapped so the Gold nanoparticles identified in both imaging modules overlap as shown in Fig. 3a for the whole imaging area of the iPALM.

To generate the overlap, the co-ordinates of the Gold nanoparticles were registered for both SEM $(x^{\prime},y^{\prime})$ and iPALM $(x,y)$ images. iPALM raw data are a collection of *X*, *Y* and *Z* coordinates, to compare with SEM image; during rendering the data are pixelated. The iPALM image obtained after rendering was given a 180° flip about the horizontal axis in the plane (about the *x* axis).

The transformation equations are:

$$ x^{\prime} = a\left( {x\cos \theta + y\sin \theta } \right) + x_{t} $$

$$ y^{\prime} = b\left( {x\sin \theta + y\cos \theta } \right) + y_{t} . $$

The above equation can be written in the matrix form as:

$$ X^{\prime} = X.{\beta}. $$

The $X^{\prime}$ matrix consists of coordinates from SEM and $X$ from iPALM.

Thus the β matrix contains the transform coefficients.

$$ \varvec{\beta} = \left( {\begin{array}{*{20}c} {\begin{array}{*{20}c} {a \cos \theta } \\ {a \sin \theta } \\ {b \cos \theta } \\ { b \sin \theta } \\ {x_{t} } \\ {y_{t} } \\ \end{array} } \\ \end{array} } \right). $$

The coefficients are obtained by running a minimization routine (linear regression):

$$ {\beta} = \left( {X^{T} X} \right)^{ - 1} X^{T} X^{\prime}. $$

We were able to produce overlap of the two images through translation and rotation operations without introducing warp operations to within 22 nm along *X* and 16 nm along *Y* across the full 30 µm × 30 µm image as shown in Fig. 3a. The error is calculated based on the offset between the positions of Gold nanoparticles after application of transformation. The error is not homogeneous with parts of the image exhibiting a larger error than the rest as shown in Figure S2.

As shown in the zoom areas in Fig. 3, the SEM images show if the iPALM data are from individual virions (Fig. 3b) and/or Dendra molecules deposited on the glass surface away from virions (Fig. 3c). The presence of Gag-Dendra molecules deposited on the glass away from the virion is not surprising since the absorption to the glass surface was performed in a non-specific method and cleaned coverslips are highly charged facilitating the binding of residual proteins in the VLP preparations to the glass coverslips. These deposits were easily distinguished through their limited height in iPALM and also lack of visible virions in SEM.

### Visualizing the position of proteins within single HIV VLPs immobilized on glass

**Fig. 4:**
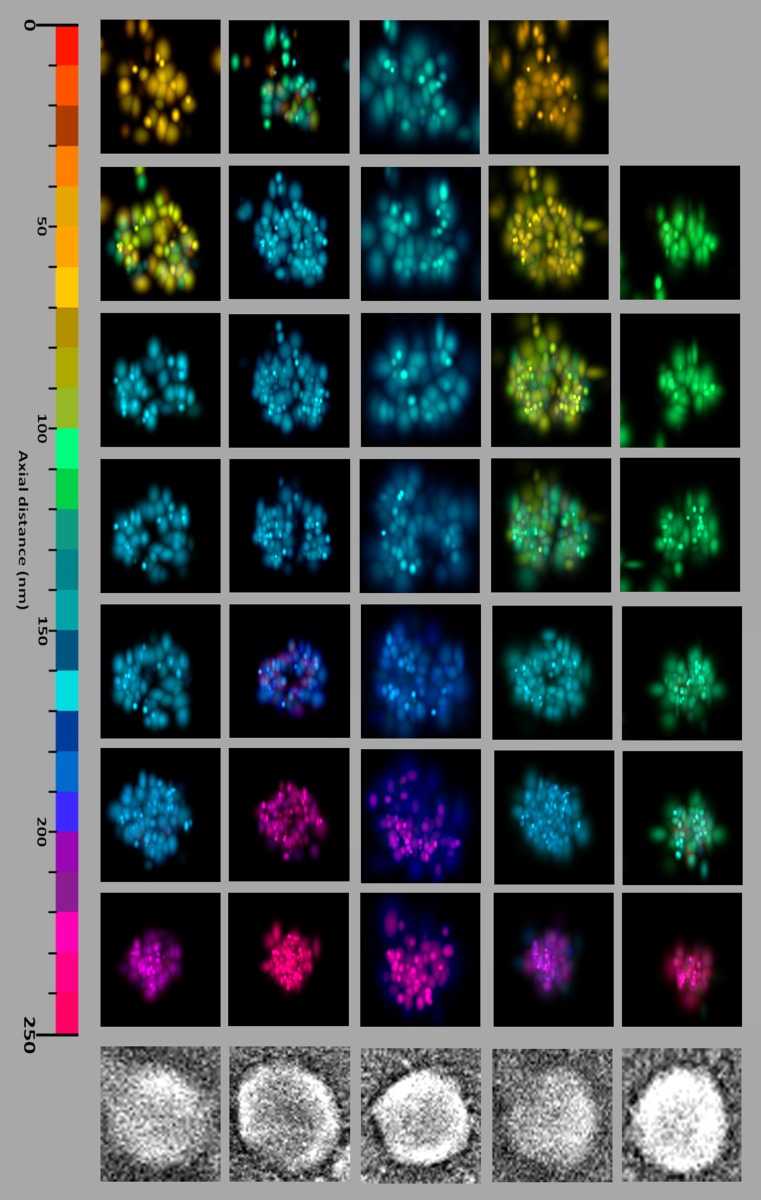
3D visualization of HIV Gag-Dendra molecules within lumen of single virions. iPALM as well as SEM images of five VLPs identified within the CLEM region shown in Fig. 3. The SEM image at the bottom corresponds to the iPALM images at the top for each VLP. From left to right the selected virions have a diameter of 170, 220, 190, 190 and 160 nm based on SEM images and contain a total of 795, 943, 797, 867 and 648 Dendra molecules based on iPALM analysis. To create a better representation the images are scaled to the same size to highlight the virion cavities and Gag Lattice defects. As explained further in the text, the diameter measured in SEM is influenced by sputtering of Gold Cadmium and therefore represents an overestimate of the VLP sizes

Once a VLP is identified in SEM along with its corresponding iPALM data, the iPALM data are plotted in 3D and represent the Gag-Dendra arrangement inside of the virus particle. Figure 4, shows the 3D distribution of Gag-Dendra within five virions selected from overlapping SEM-iPALM area shown in Fig. 3. From left to right the selected virions have a diameter of 170, 220, 190, 190 and 160 nm based on SEM images and contain a total of 795, 943, 797, 867 and 648 Dendra molecules based on iPALM analysis. To create a better representation the images are scaled to the same size to highlight the virion cavities and Gag Lattice defects.

Given the 15 nm deposition of Gold Cadmium between iPALM imaging and SEM we estimate that the reported sizes of the virions by the SEM should be corrected by the thickness of Gold Cadmium and the inevitable slight collapse of the VLPs and therefore our reported average of VLP diameters of 180 nm is an overestimate of VLP diameters before SEM.

The iPALM gallery of Dendra localization within the VLPs, shows clear areas within the virions that are void of any Dendra molecules and represent virion cavities and defects in the Gag lattice.

### Statistical analysis of the iPALM data

When middle section of the virions are imaged in iPALM, both cavity and lattice spaces void of Gag-Dendra are visible. The VLPs deposited on glass were purified from transfection of Gag-Dendra only and therefore all Gag molecules are expected to carry Dendra. However, approximately only half of the expressed Dendra molecules are actually fluorescent (Lee et al. 2012; Rollins et al. 2015). Therefore, it is reasonable to ask if the void areas within the virions are merely the artifacts of dark Dendra molecules.

As shown below however, based on the statistical calculations, the contribution of dark Dendra to observed voids is insignificant. In average we detect about 700 individual Dendra molecules within each VLP. It is helpful to calculate what would be the probability of detecting a void assuming random distribution of these molecules. The probability of having a Dendra molecule within a 10 nm^3^ voxel will be 0.003 from one assuming a 50 nm inside radius for the VLP (the probability is equal to the ratio between volume of the voxel divided by the volume of the VLP). The probability of having no Dendra molecule within this voxel is therefore 0.997 out of one. Given there are 700 Dendra molecules, having a 10 nm^3^ voxel void of any Dendra is (0.997)^700^ = 0.12 which is 12%. All voids found in the cavity of the virions as shown in the gallery in Fig. 4 are a combination of many 10 nm^3^ voxels. The probability of a void composed of *n* (10 nm^3^) voxels can be calculated as (0.12)^*n*^, therefore any void exceeding a volume encompassing two or more 10 nm^3^ voxels has a probability below 1% to be due to random positioning of Dendra molecules. This calculation is based on most generous distribution of Dendra within the volume, in reality the Dendra molecules are confined to a shell within the lattice and therefore the probability of having a void 10 nm^3^ on this shell is significantly lower.

## Discussion

Optical imaging on HIV budding and recruitment of cellular proteins has shed light on assembly kinetics of virions (Ivanchenko et al. 2009; Jouvenet et al. 2008; Ku et al. 2013) and recruitment of cellular components to the budding sites (Baumgartel et al. 2011; Jouvenet et al. 2011; Ku et al. 2014). High resolution optical microscopy has been used to localize cellular components around HIV budding sites (Bleck et al. 2014; Prescher et al. 2015; Van Engelenburg et al. 2014); however, kinetics of HIV virion release demonstrate a competition between HIV protease activation and virion release the mechanism of which remains unexplored and requires development of new imaging methodologies (Bendjennat and Saffarian 2016).

Optical imaging has always suffered from poor axial resolution compared with the resolution within the focal plane. The axial resolution was first improved by introduction of the pinhole in the scanning confocal microscopy in 1970’s (Koppel et al. 1976) and saw a major improvement by introduction of the 4Pi microscopy in 1990’s (Hell and Stelzer 1992). iPALM microscopy uses interferometry to increase the axial resolution of single molecule localization to 10 nm, which is better than the focal plane resolution of the same molecule (Shtengel et al. 2009). Here we demonstrated a methodology which allows identification of single HIV virions on glass coverslips using SEM. The localization of HIV Gag proteins within single virions are then shown with 10 nm axial and 20 nm in plane resolution. We have visualized the virion cavity along with imperfections within the HIV Gag lattice. Our observations are consistent with the previous cryo-EM measurements of immature HIV virions (Carlson et al. 2008, 2010; Schur et al. 2014).

While iPALM microscopy presents a very high overall resolution, a major caveat remains which initiates from not all Dendra molecules being localized within the sample. Almost half Dendra molecules remain in dark state either due to triplet states, improper folding or photobleaching (Lee et al. 2012; Rollins et al. 2015). Therefore at best, iPALM presents a 3D representation of 50% of the Dendra molecules within the sample. This fundamental caveat does not affect localization of high copy number proteins similar to HIV Gag; however, it will create a major challenge for localizing low copy number proteins within viral cavities. WT HIV virions incorporate ~ 2000 copies of HIV Gag within each individual virion estimated from cryo-electron tomography measurements (Carlson et al. 2008). When HIV Gag VLPs are analyzed using optical spectroscopy techniques (Chen et al. 2009) the average number of Gag molecules per virion is significantly smaller. Our measurements on HIV Gag VLPs are more consistent with the optical spectroscopy measurements and bring out the possibility that HIV Gag VLPs have a slightly less dense Gag lattice when compared with wild-type HIV virions.

We have demonstrated both the strengths and weaknesses of the SEM-iPALM CLEM using purified HIV Gag VLPs deposited on glass coverslips. However, the critical issue will be application of this technique to other viruses and or fully infectious HIV virions. Based on our analysis and the statistics presented, this method can be directly applied for creating an image of capsids which have copy numbers above ~ 1000 proteins within localized virions. However, as discussed at length in both results and discussion, direct imaging using iPALM is much less applicable for resolving the position of molecules with copy numbers below 100 since voids imaged at these copy numbers can be due to presence of dark fluorescent proteins.

How can the iPALM data then be used for proteins with copy numbers below 100. The caveat as explained above is that not all Dendra molecules will be localized within each virion. However, the molecules that are visualized are completely independent from each other. The iPALM technique can therefore report accurately the distance between pairs of observed molecules. While these distance measurements are not sufficient to resolve molecules within a single virion, performing iPALM on an ensemble of virions will result in measurement of histograms of molecular distance distributions. For simplicity, let us assume that 100 copies of a particular protein will always be clustered at one side of the virion. While the iPALM data will not be sufficient to directly image this clustering, the pairwise distance distribution of these molecules measured in an ensemble of virions will result in identification of only small distances between molecules and therefore computationally indicate the presence of clusters.

## Electronic supplementary material

Below is the link to the electronic supplementary material.

Supplementary material 1 (TIFF 2484 kb) Fig. S1: Lithography of Glass coverslips. The images show (A) zoom-out view of the mask showing the general pattern, (B) area imaged around one set of islands, (C) detailed zoom, showing the position of 5 × 5 µm islands, which are etched 250 nm deep

Supplementary material 2 (TIFF 402 kb) Fig. S2: Spatial distribution of errors in CLEM. After performing the regression algorithm and finding the best translation, rotation and scale matrix components, the position of Gold nanoparticles was analyzed and the residual difference in the position of Gold particles between SEM and iPALM is plotted as indication of error. As shown in the graph, the error in aligning SEM and iPALM is nonlinear and is more pronounced at certain positions in the sample than others (for ease of visualization, the error bars are multiplied by 10 so they become clearly visible).

## References

[CR1] Baumgartel V, Ivanchenko S, Dupont A, Sergeev M, Wiseman PW, Krausslich H-G, Brauchle C, Muller B, Lamb DC (2011). Live-cell visualization of dynamics of HIV budding site interactions with an ESCRT component. Nat Cell Biol.

[CR2] Bendjennat M, Saffarian S (2016). The race against protease activation defines the role of ESCRTs in HIV budding. PLoS Pathog.

[CR3] Bleck M, Itano MS, Johnson DS, Thomas VK, North AJ, Bieniasz PD, Simon SM (2014). Temporal and spatial organization of ESCRT protein recruitment during HIV-1 budding. Proc Natl Acad Sci.

[CR4] Briggs JAG, Simon MN, Gross I, Kräusslich H-G, Fuller SD, Vogt VM, Johnson MC (2004). The stoichiometry of Gag protein in HIV-1. Nat Struct Mol Biol.

[CR5] Briggs JAG, Riches JD, Glass B, Bartonova V, Zanetti G, Kräusslich H-G (2009). Structure and assembly of immature HIV. Proc Natl Acad Sci.

[CR6] Carlson L-A, Briggs JAG, Glass B, Riches JD, Simon MN, Johnson MC, Müller B, Grünewald K, Kräusslich H-G (2008). Three-dimensional analysis of budding sites and released virus suggests a revised model for HIV-1 morphogenesis. Cell Host Microbe.

[CR7] Carlson L-A, de Marco A, Oberwinkler H, Habermann A, Briggs JAG, Kräusslich H-G, Grünewald K (2010). Cryo electron tomography of native HIV-1 budding sites. PLoS Pathog.

[CR8] Chen Y, Wu B, Musier-Forsyth K, Mansky LM, Mueller JD (2009). Fluorescence fluctuation spectroscopy on viral-like particles reveals variable gag stoichiometry. Biophys J.

[CR9] Gheysen D, Jacobs E, de Foresta F, Thiriart C, Francotte M, Thines D, De Wilde M (1989). Assembly and release of HIV-1 precursor Pr55gag virus-like particles from recombinant baculovirus-infected insect cells. Cell.

[CR10] Hell S, Stelzer EHK (1992). Properties of a 4pi confocal fluorescence microscope. J Opt Soc Am A Opt Image Sci Vis.

[CR11] Hess ST, Girirajan TPK, Mason MD (2006). Ultra-high resolution imaging by fluorescence photoactivation localization microscopy. Biophys J.

[CR12] Hodges JA, Saffarian S (2014). Sample preparation for single virion atomic force microscopy and super-resolution fluorescence imaging. JoVE.

[CR13] Hodges J, Tang X, Landesman MB, Ruedas JB, Ghimire A, Gudheti MV, Perrault J, Jorgensen EM, Gerton JM, Saffarian S (2013). Asymmetric packaging of polymerases within vesicular stomatitis virus. Biochem Biophys Res Commun.

[CR14] Huang B, Wang W, Bates M, Zhuang X (2008). Three-dimensional super-resolution imaging by stochastic optical reconstruction microscopy. Science.

[CR15] Ivanchenko S, Godinez WJ, Lampe M, Kräusslich H-G, Eils R, Rohr K, Bräuchle C, Müller B, Lamb DC (2009). Dynamics of HIV-1 assembly and release. PLoS Pathog.

[CR16] Jouvenet N, Bieniasz PD, Simon SM (2008). Imaging the biogenesis of individual HIV-1 virions in live cells. Nature.

[CR17] Jouvenet N, Zhadina M, Bieniasz PD, Simon SM (2011). Dynamics of ESCRT protein recruitment during retroviral assembly. Nat Cell Biol.

[CR18] Juette MF, Gould TJ, Lessard MD, Mlodzianoski MJ, Nagpure BS, Bennett BT, Hess ST, Bewersdorf J (2008). Three-dimensional sub-100 nm resolution fluorescence microscopy of thick samples. Nat Methods.

[CR19] Kaksonen M, Drubin DG (2006). PALM reading: seeing the future of cell biology at higher resolution. Dev Cell.

[CR20] Kofman A, Graf M, Bojak A, Deml L, Bieler K, Kharazova A, Wolf H, Wagner R (2003). HIV-1 gag expression is quantitatively dependent on the ratio of native and optimized codons. Tsitologiia.

[CR21] Kopek BG, Paez-Segala MG, Shtengel G, Sochacki KA, Sun MG, Wang Y, Xu CS, van Engelenburg SB, Taraska JW, Looger LL, Hess HF (2017). Diverse protocols for correlative super-resolution fluorescence imaging and electron microscopy of chemically fixed samples. Nat Protoc.

[CR22] Koppel DE, Axelrod D, Schlessinger J, Elson EL, Webb WW (1976). Dynamics of fluorescence marker concentration as a probe of mobility. Biophys J.

[CR23] Ku P-I, Miller Anna K, Ballew J, Sandrin V, Adler Frederick R, Saffarian S (2013). Identification of pauses during formation of HIV-1 virus like particles. Biophys J.

[CR24] Ku P-I, Bendjennat M, Ballew J, Landesman MB, Saffarian S (2014). ALIX is recruited temporarily into HIV-1 budding sites at the end of gag assembly. PLoS ONE.

[CR25] Lee S-H, Shin JY, Lee A, Bustamante C (2012). Counting single photoactivatable fluorescent molecules by photoactivated localization microscopy (PALM). Proc Natl Acad Sci.

[CR26] Prescher J, Baumgärtel V, Ivanchenko S, Torrano AA, Bräuchle C, Müller B, Lamb DC (2015). Super-resolution imaging of ESCRT-proteins at HIV-1 assembly sites. PLoS Pathog.

[CR27] Rollins GC, Shin JY, Bustamante C, Pressé S (2015). Stochastic approach to the molecular counting problem in superresolution microscopy. Proc Natl Acad Sci.

[CR28] Rust MJ, Bates M, Zhuang XW (2006). Sub-diffraction-limit imaging by stochastic optical reconstruction microscopy (STORM). Nat Methods.

[CR29] Schur FKM, Hagen WJH, Rumlova M, Ruml T, Muller B, Krausslich H-G, Briggs JAG (2014). Structure of the immature HIV-1 capsid in intact virus particles at 8.8 Å resolution. Nature.

[CR30] Shtengel G, Galbraith JA, Galbraith CG, Lippincott-Schwartz J, Gillette JM, Manley S, Sougrat R, Waterman CM, Kanchanawong P, Davidson MW, Fetter RD, Hess HF (2009). Interferometric fluorescent super-resolution microscopy resolves 3D cellular ultrastructure. Proc Natl Acad Sci.

[CR31] Thompson RE, Larson DR, Webb WW (2002). Precise nanometer localization analysis for individual fluorescent probes. Biophys J.

[CR32] Van Engelenburg SB, Shtengel G, Sengupta P, Waki K, Jarnik M, Ablan SD, Freed EO, Hess HF, Lippincott-Schwartz J (2014). Distribution of ESCRT machinery at HIV assembly sites reveals virus scaffolding of ESCRT subunits. Science.

