# Supplementary figures and images for: Correlative iPALM and SEM resolves virus cavity and Gag lattice defects in HIV virions
Source: Eur Biophys J. 2018 Jul 24;48(1):15–23. doi: 10.1007/s00249-018-1324-0 (PMC6330563; doi:10.1007/s00249-018-1324-0)

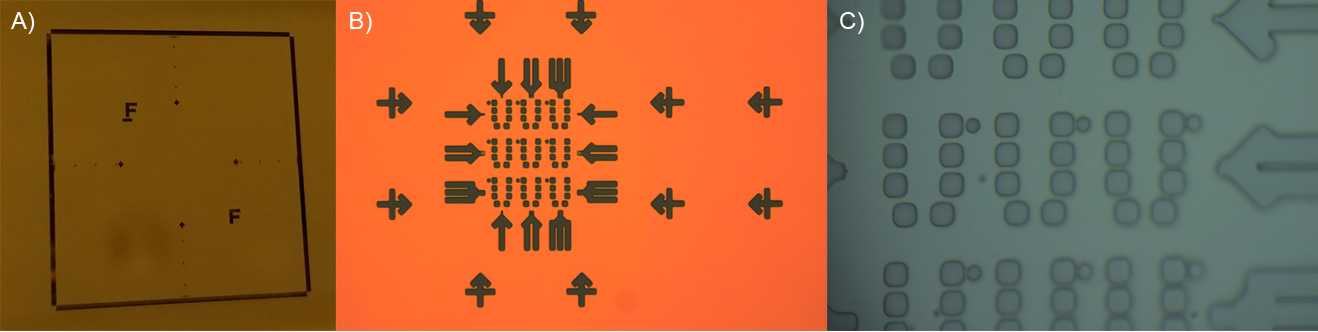

Supplement: Supplementary file 1 — Supplementary material 1 (TIFF 2484 kb) Fig. S1: Lithography of Glass coverslips. The images show (A) zoom-out view of the mask showing the general pattern, (B) area imaged around one set of islands, (C) detailed zoom, showing the position of 5 × 5 µm islands, which are etched 250 nm deep [file 249_2018_1324_MOESM1_ESM.tif]
